# The Baby

**Published:** 1874-01

**Authors:** 


### THE BABY.

Wife says it invariably happens that when editors exhibit a propensity for writing short articles upon babies, that there is sure to be a little visitor in said editors' mansions, within a reasonably short time. But, in this particular instance, her prediction must fail, for we *have* a baby; a little blue-eyed rogue, who is this blessed minute affording us the sprightly entertainment of being "put to sleep."—Gracious! do you know what that formula means ? Ever see a mother bundle a baby in flannels, until it resembled an Egyptian mummy,—grasp him lovingly in her arms, hop from the bed, at the dead of night, and dart for the register in adjoining room, to trot and sing the little rascal to sleep ! Ever see that performance ? It is ridiculously funny ! Our baby is at that interesting age in which they are most prone to colic. Why all babies are made up with that exasperating accompaniment, is a mystery beyond our ken. They all have it, and cry and scream like fury, until you are on the verge of desperation in seeking means of relief. Having a doctor in the house, we are spared the usual procedure of seeking him elsewhere,—but the colic is just as bad as though his abiding place was as remote as other people's physician. Now, we never *did* know precisely, what to do with a baby in colic, particularly our *own* baby ; although, in our quiet moments, in the retirement of our study, we could give a visiting mother capital advice upon the management of a colicy baby, provided she came unaccompanied by the unfortunate child.—But the moment that scream reaches your ear, and the upturned heels of the baby, perchance, caroms upon your nose; while mother, aunty and grandma, are administering peppermint, soothing syrup, paregoric, catnip tea, camphor-water, and hot fomentations, we are so confused and perplexed at the turmoil incident to all this performance, as to be in doubt as to the propriety of further interference, or whether any room could be found in its stomach, or anywhere about its person, for that matter, for any scientific attack upon the suffering urchin.

Then, how wondrously strange it is, that all babies should have a special hour for this gymnastic exercise. I say *all*, for fathers agree, with remarkable unanimity, that 4 o'clock A. M., is the precise moment of attack. Why it could not occur at 4 P. M., for instance, or about the time your wife is preparing to make a shopping excursion in search of the last new bonnet, or even at 10 o'clock P. M.,—just before you take your departure from the Club, is more than we can conjecture. That 4 A. M., is almost certain to catch a fellow napping; for then, you know, no reasonable man can have any plausable business down town; and this fact is extremely harassing.

Sometimes the experiment of rocking the baby is attempted in bed, the mother resolving her person into a cradle, bobbing up and down, upon your spring bed, like a rubber ball. This soothing process acts admirably upon the child, but is slightly perplexing to the sleepless father. It tends to shatter your confidence in the law of gravitation, particularly when the mother of your child is of considerable more pounds, avoirdupois, than yourself; for, when they go down, somehow, you go up, and the uncertainty of alighting always in the same spot is so noteworthy as to establish in your breast more thoughts of your own comfort and safety, than for that of the blessed baby.

Now, dear reader, perhaps you think these reflections are not the result of actual experience.—Ask your married friend, and, if he is the delighted father of a new baby, watch him three months hence. He will not salute you so gayly when you meet him in the morning. He will yawn and rub his eyes at noontime, and will pass you unnoticed by evening;—his baby had the colic last night, ask him about it, you will enjoy the story, and we shall be delighted with his company.

